# Association between sarcopenic obesity, obesity, sarcopenia and quality of life in middle-aged and older Chinese: the Guangzhou Biobank Cohort Study

**DOI:** 10.1007/s11136-025-03960-9

**Published:** 2025-04-02

**Authors:** Jing Yi Chen, Jiao Wang, Ya Li Jin, Kar Keung Cheng, Tai Hing Lam, Wei Sen Zhang, Lin Xu

**Affiliations:** 1 School of Public Health, Sun Yat-sen University, Guangzhou, China; 2 Guangzhou Twelfth People’s Hospital, Guangzhou, China; 3 Institute of Applied Health Research, University of Birmingham, Birmingham, UK; 4 School of Public Health, the University of Hong Kong, Hong Kong, China; 5 Greater Bay Area Public Health Research Collaboration, Guangzhou, China

**Keywords:** Older people, Obesity, Sarcopenia, Sarcopenic obesity, Quality of life

## Abstract

**Purpose:**

Sarcopenic obesity (SO) is increasing globally, especially in aging populations. This study aims to analyze whether SO is more strongly associated with poorer quality of life (QoL) than obesity or sarcopenia alone.

**Methods:**

SO was defined as the coexistence of probable sarcopenia combined with obesity. Obesity was defined by body mass index and waist circumference, and probable sarcopenia was identified using the Asian Working Group for Sarcopenia criteria 2019. QoL was assessed using the Short-Form 12 Health Survey Version 2. Linear regression was used to analyze the association between SO with QoL composite and domain scores.

**Results:**

Of 6,332 participants aged 50 years or older, 21.9% had SO, 10.6% had obesity only, and 38.7% had probable sarcopenia only. Compared to participants without obesity or sarcopenia, those with SO showed a significant negative association with physical health-related QoL (all *P* < 0.05), which remained significant when obesity or sarcopenia alone was used as the reference. The adjusted beta (95% CI) for the Physical Component Summary, General Health, Physical Functioning, Role Physical, and Bodily Pain for the SO group was -1.23 (-1.68, -0.79), -0.86 (-1.51, -0.21), -1.28 (-1.77, -0.80), -0.51 (-0.95, -0.07), and -0.77 (-1.37, -0.18), respectively. Non-significant association of SO with the Mental Component Summary, Vitality, Role Limitation due to Emotional Problem, and Mental Health was found (all *P* > 0.05). The results were consistent across different SO criteria and by sex (*P* for sex-interaction from 0.21 to 0.99).

**Conclusion:**

SO was associated with lower physical health-related QoL compared to obesity or sarcopenia alone, but non-significantly associated with mental health-related QoL.

**Supplementary Information:**

The online version contains supplementary material available at 10.1007/s11136-025-03960-9.

## Introduction

Sarcopenic obesity (SO) is complex phenotype that combines excess adiposity with a significant reduction in muscle mass and strength [1, 2]. The clinical significance of SO is underscored by its association with more severe adverse health outcomes compared to obesity or sarcopenia alone, including higher risks of all-cause mortality, cardiovascular disease, metabolic syndrome, and type 2 diabetes [3–7]. A recent systematic review and meta-analysis showed that over a tenth of older adults globally were affected by SO [8]. With the global population aging, the prevalence of SO is anticipated to increase, highlighting the need for healthcare systems and policymakers to understand and address far-reaching implications of SO.

The coexistence of obesity and sarcopenia in SO complicates the clinical management of affected individuals, often exacerbating declines in physical function and overall well-being. These deteriorations are closely tied to quality of life (QoL), a multifaceted construct including physical, psychological, and social domains of health. However, the association between SO and QoL remains inconclusive. Some studies reported that individuals with SO experienced poorer QoL compared to those without obesity or sarcopenia, or those with obesity alone [9–12]. However, some studies found no significant differences or suggested that SO may have better QoL than those with sarcopenia alone [10, 13]. Given the conflicting evidence and the importance of this issue, our study aimed to analyze the association between SO and QoL in middle-aged and older Chinese adults, using data from the Guangzhou Biobank Cohort Study (GBCS).

## Methods

### Study sample

All participants were recruited from the GBCS, details which have been reported previously [14]. Briefly, GBCS is a 3-way collaboration among the Guangzhou Twelfth People’s Hospital and the Universities of Hong Kong, China, and Birmingham, UK. Participants were recruited from the “Guangzhou Health and Happiness Association for the Respectable Elders (GHHARE)”, a community social and welfare organization. Membership was open to Guangzhou permanent residents aged 50 years or older for a nominal fee of 4 CNY (≈ 50 US cents) per month. Baseline information collected by a face-to-face, computer-assisted interview by trained nurses included demographic characteristics, lifestyle factors, family, and personal medical history. Anthropometric parameters, blood pressure, fasting plasma glucose, lipids, and inflammatory markers were measured. The reliability of the questionnaire was tested by recalling 200 randomly selected participants for re-interview, and the results were satisfactory [14]. The Guangzhou Medical Ethics Committee of the Chinese Medical Association approved the study, and all participants gave written, informed consent before participation. We used baseline data from GBCS from September 2006 to 2007. Participants with cancer were excluded.

### Exposures

Probable sarcopenia was defined according to the 2019 Asian Working Group for Sarcopenia (AWGS 2019) criteria, characterized by grip strength below 28 kg in men and 18 kg in women, or a timed up-and-go test (TUGT) exceeding 5 s, or both [15]. Grip strength was measured by a Jamar Hydraulic Hand Dynamometer in a standing position, a method known for its high test-retest reproducibility (*r* > 0.80) [16] and excellent interrater reliability (*r* = 0.98) [17]. The maximum grip strength value obtained, regardless of the dominant hand or number of tests, was recorded as per AWGS 2019 guidelines [15]. The TUGT involved participants rising from a chair, walking 2.5 m around a marker, and returning. Each test was timed by nurses and performed twice, with the average score used for analysis. These data had been used in several GBCS papers [18, 19].

General obesity was identified with a body mass index (BMI) ≥ 28 kg/m^2^, in accordance with the criteria set by the National Health and Family Planning Commission of the People’s Republic of China [20]. For sensitivity analyses, an alternative BMI cutoff of ≥ 30 kg/m^2^, as recommended by WHO guidelines, was also applied [21]. Abdominal obesity was defined by a waist circumference (WC) ≥ 90 cm for men or ≥ 80 cm for women [22].

Sarcopenic obesity (SO) was defined as the coexistence of probable sarcopenia with either general or abdominal obesity (Criteria 1 in Supplementary Table 1). For sensitivity analyses, four alternative SO criteria incorporating different probable sarcopenia indicators, obesity indicators and BMI thresholds were used (Supplementary Table 1).

Participants were categorized into four groups based on their obesity and sarcopenia status: (1) neither obesity nor sarcopenia (NONS), (2) isolated obesity (IO), (3) isolated sarcopenia (IS), and (4) sarcopenic obesity (SO).

### Outcomes

The primary outcome was quality of life (QoL), assessed using the Short-Form 12 Health Survey Version 2 (SF-12v2). This instrument includes 12 items covering eight domains of health-related quality of life: physical functioning (PF), role limitation due to physical problems (RP), bodily pain (BP), general health (GH), vitality (VT), social functioning (SF), role limitation due to emotional problem (RE), and mental health (MH) [23]. From these domains, two composite scores were calculated: the Physical Component Summary (PCS) and the Mental Component Summary (MCS) [23]. To facilitate straightforward interpretation, the scores were standardized to US population norms (mean = 50, standard deviation = 10) with higher scores indicating better QoL [23].

### Potential confounders

Potential confounders considered in the analysis included sex, age (years), education levels (primary or below, secondary, and college or above), occupation (manual, non-manual, and others), annually family income (< 10,000, 10,000–29,999, 30,000–49,999, ≥ 50,000 CNY/year, and don’t know). Physical activity was classified as inactive, moderate, and active based on established criteria [24]. Dietary patterns were assessed using quartiles of adherence to the Dietary Approaches to Stop Hypertension (DASH) diet [25]. Smoking status was categorized as never, former, or current smokers, while alcohol use was classified as never, former, or current drinkers [26]. Additional confounders included sleep duration (7–8, < 7 and ≥ 9 h/day), snoring status (no, yes, and don’t know), the number of falls in past 6 months (0, and ≥ 1), and the 15-item Geriatric Depression Scale (GDS-15) score [27]. The number of co-morbidities was grouped as ≤ 1, 2, or ≥ 3, with co-morbidities including 18 diseases: hypertension, dyslipidemia, type 2 diabetes mellitus, coronary heart disease, stroke, angina, rheumatic heart disease, arrhythmia, liver disease, gastrointestinal disease, chest disease, genitourinary disease, neurological disease, eye disease, arthritis, thyroid disease, fracture history, and mental disease.

### Statistical analysis

Continuous variables were summarized as mean (standard deviation (SD)) for normally distributed data, or as median (inter-quartile range (IQR)) for skewed data. Categorical variables were reported as numbers (percentages). Chi-square tests and one-way analysis of variance (ANOVA) were used to compare sample characteristics across the four SO groups. Multivariable linear regression with adjustment for sex, age, family income, education, occupation, smoking status, alcohol use, DASH diet adherence, snoring, physical activity, number of co-morbidities, number of falls in past 6 months, and GDS-15 score, was used to analyze the association between the SO groups with QoL composite and domain scores, using the NONS as the reference group, yielding adjusted regression coefficients (β) and 95% confidence intervals (CI). Additionally, QoL comparisons were made between SO and IO or IS, using multivariable linear regression with the same adjustment, with IO and IS as the reference groups, respectively. Given the observed sex differences in muscle strength and physical performance, we tested for interactions between sex and SO on QoL composite and domain scores, and conducted stratification analysis by sex. Sensitivity analyses were performed to evaluate the robustness of our finding using four alternative criteria of SO (Supplementary Table 1). All statistical analyses were conducted using Stata version 18.0 (STATA Corp LP, TX, USA) and R program version 4.3.2 (ST Louis, MO, USA). All tests were two-sided, and a P-value of < 0.05 was considered statistically significant.

## Results

Of the 9,801 participants who completed the SF-12v2 questionnaire, 3,469 were excluded due to incomplete information on TUGT, grip strength, BMI and WC, or extreme values of TUGT and grip strength, leaving 6,332 participants (75.3% women) in the present study. Of them, 3,842 (60.7%) were identified as probable sarcopenia: 578 (9.1%) based on grip strength only, 2,222 (35.1%) based on TUGT only, and 1,042 (16.5%) meeting both criteria. Additionally, 680 (10.7%) participants had a BMI ≥ 28 kg/m^2^, 248 (3.9%) had a BMI ≥ 30 kg/m^2^, and 1,985 (31.4%) were abdominal obesity (Supplementary Table 1). Based on the classification criteria 1, 1,819 (28.7%) participants were in the NONS group, 671 (10.6%) in the IO group, 2,453 (38.7%) in the IS group, and 1,389 (21.9%) in the SO group. The mean age of participants was 60.45 (standard deviation (SD) = 7.7) years (Table 1).

**Table 1:** Characteristics of 6,332 participants in the Guangzhou Biobank Cohort Study by sarcopenic obesity groups

	Total	SO groups				*P* value
		NONS	IO	IS	SO	
**Number of participants**,** N (%)**	6,332 (100.0)	1,819 (28.7)	671 (10.6)	2,453 (38.7)	1,389 (21.9)	-
**Sex**,** N (%)**						< 0.001
women	4,771 (75.3)	1,253 (68.9)	573 (85.4)	1,744 (71.1)	1,201 (86.5)	
men	1,561 (24.7)	566 (31.1)	98 (14.6)	709 (28.9)	188 (13.5)	
**Age**,** years**,** mean (SD)**	60.45 (7.7)	57.34 (5.6)	57.60 (5.8)	61.90 (8.2)	63.33 (8.0)	< 0.001
**Education**,** N (%)**						< 0.001
≤primary	2,403 (38.0)	365 (20.1)	247 (36.8)	1,003 (40.9)	788 (56.7)	
middle school	3,374 (53.3)	1,245 (68.4)	369 (55.0)	1,249 (50.9)	511 (36.8)	
≥college	555 (8.8)	209 (11.5)	55 (8.2)	201 (8.2)	90 (6.5)	
**Occupation**,** N (%)**						< 0.001
manual	3873 (61.7)	971 (53.9)	424 (64.3)	1531 (62.8)	947 (68.7)	
non-manual	1293 (20.6)	485 (26.9)	120 (18.2)	472 (19.4)	216 (15.7)	
other	1,112 (17.7)	346 (19.2)	115 (17.5)	435 (17.8)	216 (15.7)	
**Family income**,** CNY/year**,** N (%)**						< 0.001
< 10,000	303 (4.8)	52 (2.9)	19 (2.8)	132 (5.4)	100 (7.2)	
10,000–29,999	2,038 (32.2)	541 (29.8)	208 (31.0)	799 (32.6)	490 (35.3)	
30,000–49,999	1,683 (26.6)	545 (30.0)	183 (27.3)	654 (26.7)	301 (21.7)	
≥ 50,000	1,335 (21.1)	476 (26.2)	160 (23.8)	458 (18.7)	241 (17.4)	
Don’t know	968 (15.3)	204 (11.2)	101 (15.1)	406 (16.6)	257 (18.5)	
**Physical activity**,** N (%)**						< 0.01
Inactive	424 (6.7)	113 (6.2)	36 (5.4)	181 (7.4)	94 (6.8)	
Moderate	1,796 (28.4)	463 (25.5)	180 (26.8)	721 (29.4)	432 (31.1)	
Active	4,112 (64.9)	1,243 (68.3)	455 (67.8)	1,551 (63.2)	863 (62.1)	
**DASH diet adherence**,** N (%)**						0.01
1st quartile	1,771 (28.0)	539 (29.6)	175 (26.1)	664 (27.1)	393 (28.3)	
2nd quartile	2,003 (31.6)	577 (31.7)	218 (32.5)	747 (30.5)	461 (33.2)	
3rd quartile	1,117 (17.6)	273 (15.0)	134 (20.0)	454 (18.5)	256 (18.4)	
4th quartile	1,441 (22.8)	430 (23.6)	144 (21.5)	588 (24.0)	279 (20.1)	
**Smoking status**,** N (%)**						< 0.001
Never	5,241 (82.8)	1,475 (81.1)	602 (89.9)	1,947 (79.4)	1,217 (87.8)	
Former	473 (7.5)	141 (7.8)	34 (5.1)	212 (8.6)	86 (6.2)	
Current	613 (9.7)	203 (11.2)	34 (5.1)	293 (11.9)	83 (6.0)	
**Alcohol use**,** N (%)**						< 0.001
Never	1,719 (33.8)	428 (28.1)	181 (32.7)	668 (34.4)	442 (41.2)	
Former	196 (3.9)	46 (3.0)	26 (4.7)	71 (3.7)	53 (4.9)	
Current	3,175 (62.4)	1,047 (68.8)	347 (62.6)	1,202 (61.9)	579 (53.9)	
**Sleep duration**,** hours/day**,** N (%)**						< 0.01
7–8	3,311 (52.3)	1,024 (56.3)	346 (51.6)	1,246 (50.8)	695 (50.0)	
< 7	2,443 (38.6)	643 (35.3)	261 (38.9)	984 (40.1)	555 (40.0)	
≥ 9	578 (9.1)	152 (8.4)	64 (9.5)	223 (9.1)	139 (10.0)	
**Snoring**,** N (%)**						0.05
No	2,086 (32.9)	588 (32.3)	174 (25.9)	925 (37.7)	399 (28.7)	
Yes	2,779 (43.9)	805 (44.3)	353 (52.6)	951 (38.8)	670 (48.2)	
Don’t know	1,467 (23.2)	426 (23.4)	144 (21.5)	577 (23.5)	320 (23.0)	
**Number of falls in past 6 months**,** N (%)**						< 0.01
0	5,914 (93.5)	1,719 (94.7)	627 (93.4)	2,285 (93.3)	1,283 (92.4)	
≥ 1	411 (6.5)	96 (5.3)	44 (6.6)	165 (6.7)	106 (7.6)	
**GDS-15 score**,** mean (SD)**	2.3 (2.2)	2.0 (2.0)	2.2 (2.2)	2.4 (2.3)	2.5 (2.4)	< 0.001
**Number of co-morbidities**,** N (%)**						< 0.001
0–1	2,322 (36.7)	748 (41.1)	257 (38.3)	885 (36.1)	432 (31.1)	
2	1,578 (24.9)	418 (23.0)	146 (21.8)	656 (26.7)	358 (25.8)	
≥ 3	2,432 (38.4)	653 (35.9)	268 (39.9)	912 (37.2)	599 (43.1)	
**BMI**,** mean (SD)**	24.1 (3.1)	22.7 (2.1)	26.7 (2.8)	22.8 (2.2)	27.0 (2.9)	< 0.001

NONS = neither obesity nor sarcopenia, IO = isolated obesity, IS = isolated sarcopenia, SO = sarcopenic obesity, SD = standard deviation, CNY = Chinese yuan, GDS-15 = The 15-item Geriatric Depression Scale, BMI = Body mass index

Table 1 shows that, compared to participants with NONS, those with SO were older and had a higher proportion of women (all *P* < 0.001). The SO group also had lower education, a higher prevalence of manual occupation, lower family income, lower physical activity levels, lower adherence to the DASH diet pattern, and lower rates of smoking and alcohol consumption (all *P* < 0.05). Moreover, compared to the NONS group, those with SO had longer sleep duration, a greater number of co-morbidities, more falls in the past 6 months, higher GDS-15 scores, and higher BMI (all *P* < 0.01). The mean (SD) scores for the PCS and MCS, as well as the eight domains of the SF-12v2 were as follows: PCS 48.6 (5.5), MCS 55.8 (6.9), GH 32.5 (7.9), PF 53.8 (5.8), RP 55.0 (5.5), BP 53.9 (7.3), RE 53.2 (6.3), MH 55.7 (7.9), VT 58.6 (7.3) and SF 54.4 (5.8). Compared to participants with NONS, those with SO had significantly lower scores in PCS, GH, PF, RP, BP, RE, VT, and SF (all *P* < 0.01) (Table 2).

**Table 2:** SF-12v2 scores of 6,332 participants in the Guangzhou Biobank Cohort Study by sarcopenic obesity groups

SF-12v2 scores	Total	SO groups				*P* value
		NONS	IO	IS	SO	
	Mean (SD)	Mean (SD)	Mean (SD)	Mean (SD)	Mean (SD)	
**Two composite scores**						
PCS	48.6 (5.5)	49.5 (4.9)	48.9 (5.1)	48.4 (5.4)	47.4 (6.1)	< 0.001
MCS	55.8 (6.9)	55.9 (6.8)	55.9 (6.7)	55.7 (7.0)	55.7 (7.1)	0.76
**Eight domains**						
GH	32.5 (7.9)	33.5 (8.0)	32.7 (7.7)	31.9 (7.7)	31.8 (7.8)	< 0.001
PF	53.8 (5.8)	54.7 (4.6)	54.1 (5.8)	53.8 (5.6)	52.4 (6.9)	< 0.001
RP	55.0 (5.5)	55.4 (4.9)	55.2 (5.2)	55.0 (5.4)	54.3 (6.2)	< 0.001
BP	53.9 (7.3)	54.6 (6.4)	54.5 (6.4)	53.8 (7.6)	53.0 (8.1)	< 0.001
RE	53.2 (6.3)	53.5 (5.9)	53.5 (6.0)	53.1 (6.4)	52.7 (6.6)	< 0.001
MH	55.7 (7.9)	55.9 (7.7)	55.7 (7.9)	55.7 (8.0)	55.5 (8.1)	0.59
VT	58.6 (7.3)	59.1 (7.1)	58.9 (7.2)	58.4 (7.3)	58.4 (7.3)	< 0.01
SF	54.4 (5.8)	55.0 (5.0)	54.5 (5.9)	54.4 (5.8)	53.8 (6.5)	< 0.001

NONS = neither obesity nor sarcopenia, IO = isolated obesity, IS = isolated sarcopenia, SO = sarcopenic obesity, SD = standard deviation, PCS = physical component summary, MCS = mental component summary, GH = general health, PF = physical functioning, RP = role limitation due to physical problems, BP = bodily pain, RE = role limitation due to emotional problem, MH = mental health, VT = vitality, SF = social functioning

**Fig. 1:**
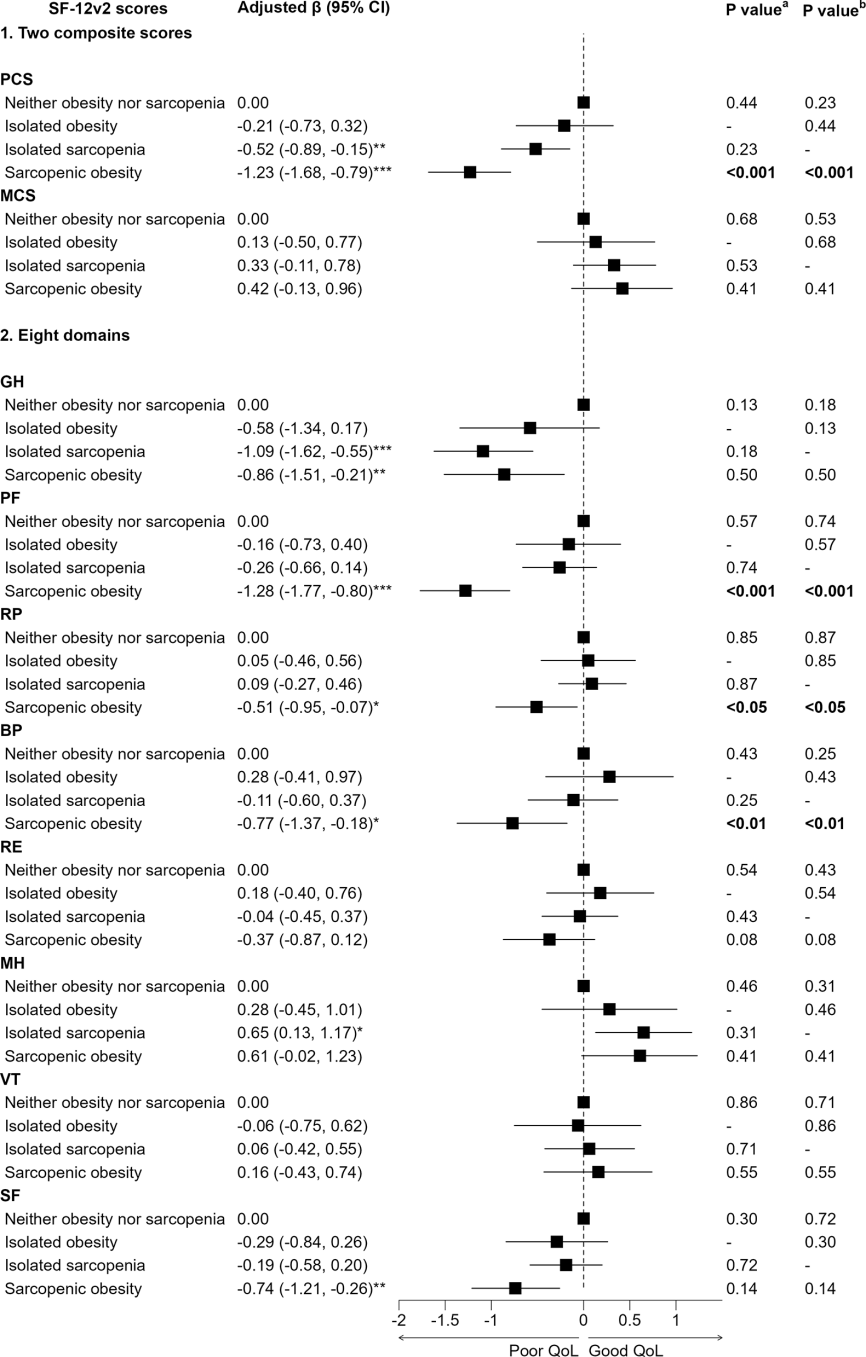
Association of sarcopenic obesity with quality of life composite and domain scores on 6,332 participants of the Guangzhou Biobank Cohort Study. Note: PCS = physical component summary, MCS = mental component summary, GH = general health, PF = physical functioning, RP = role limitation due to physical problems, BP = bodily pain, RE = role limitation due to emotional problem, MH = mental health, VT = vitality, SF = social functioning. Adjusted β (95% CI): adjusted for sex, age, family income, education, occupation, smoking status, alcohol use, DASH diet adherence, snoring, physical activity, number of co-morbidities, number of falls in past 6 months, and GDS-15 score, with neither obesity nor sarcopenia as the reference. *: *P* < 0.05, **: *P* < 0.01, ***: *P* < 0.001. **a**: with the isolated obesity as the reference. **b**: with the isolated sarcopenia as the reference

In Fig. 1, after adjusting for sex, age, family income, education, occupation, smoking status, alcohol use, DASH diet adherence, snoring, physical activity, number of co-morbidities, number of falls in past 6 months, and GDS-15 score, compared to participants with NONS, those with SO had significantly lower PCS, GH, PF, RP, BP, and SF scores. The adjusted βs (95% CIs) were as follows: PCS -1.23 (-1.68, -0.79), GH -0.86 (-1.51, -0.21), PF -1.28 (-1.77, -0.80), RP -0.51 (-0.95, -0.07), BP -0.77 (-1.37, -0.18), and SF -0.74 (-1.21, -0.26). Moreover, when compared to participants with IO or IS, those with SO had significantly lower PCS, PF, RP, and BP scores (all *P* < 0.05). No significant association of SO with MCS, RE, MH, or VT score was found. Participants with IS had significantly lower PCS (adjusted β -0.52, 95% CI -0.89 to -0.15) and GH (-1.09, -1.62 to -0.55) scores, and significantly higher MH scores (0.65, 0.13 to 1.17) (Fig. 1).

No significant sex-interaction was found on QoL (Table 3, *P* for interaction from 0.21 to 0.99). In sex-stratified analyses, the negative association between SO and PCS scores remained significant for both women and men. However, in women, the negative association of SO with GH (adjusted β -0.77, 95% CI -1.50 to -0.04), PF (-1.40, -1.98 to -0.82), and SF (-0.77, -1.33 to -0.21) scores remained significant (Table 3), while in men, the association between SO and RP remained significant (-1.09, -1.97 to -0.22) (Table 3).

**Table 3:** Associations of sarcopenic obesity with quality of life composite and domain scores in 4,771 women and 1,561 men of the Guangzhou Biobank Cohort Study

	Women, adjusted β (95% CI)				Men, adjusted β (95% CI)				*P* for interaction
	NONS	IO	IS	SO	NONS	IO	IS	SO	
**Two composite scores**									
PCS	0.00	-0.24 (-0.85, 0.37)	-0.55 (-1.01, -0.08)*	-1.22 (-1.75, -0.69)***	0.00	-0.17 (-1.27, 0.92)	-0.49 (-1.09, 0.10)	-1.33 (-2.22, -0.44)**	0.59
MCS	0.00	0.31 (-0.41, 1.03)	0.57 (0.02, 1.13)*	0.53 (-0.10, 1.16)	0.00	-0.21 (-1.62, 1.19)	-0.10 (-0.86, 0.67)	0.49 (-0.64, 1.63)	0.29
**Eight domains**									
GH	0.00	-0.43 (-1.27, 0.40)	-0.89 (-1.53, -0.25)**	-0.77 (-1.50, -0.04)*	0.00	-1.10 (-2.93, 0.73)	-1.50 (-2.50, -0.51)**	-1.08 (-2.56, 0.41)	0.21
PF	0.00	-0.27 (-0.93, 0.40)	-0.26 (-0.77, 0.24)	-1.40 (-1.98, -0.82)***	0.00	0.18 (-0.94, 1.30)	-0.26 (-0.87, 0.35)	-0.77 (-1.67, 0.14)	0.25
RP	0.00	0.04 (-0.55, 0.64)	0.14 (-0.31, 0.60)	-0.34 (-0.86, 0.18)	0.00	0.17 (-0.91, 1.25)	-0.05 (-0.64, 0.53)	-1.09 (-1.97, -0.22)*	0.73
BP	0.00	0.40 (-0.41, 1.21)	-0.14 (-0.77, 0.48)	-0.69 (-1.40, 0.02)	0.00	-0.33 (-1.70, 1.05)	-0.06 (-0.80, 0.69)	-0.78 (-1.89, 0.33)	0.44
RE	0.00	0.24 (-0.43, 0.92)	0.05 (-0.47, 0.57)	-0.20 (-0.79, 0.39)	0.00	0.07 (-1.12, 1.27)	-0.20 (-0.85, 0.45)	-0.71 (-1.68, 0.26)	0.60
MH	0.00	0.53 (-0.29, 1.36)	0.97 (0.33, 1.60)**	0.66 (-0.07, 1.38)	0.00	-0.45 (-2.10, 1.20)	0.02 (-0.88, 0.91)	1.13 (-0.21, 2.46)	0.54
VT	0.00	0.10 (-0.67, 0.87)	0.20 (-0.39, 0.79)	0.26 (-0.42, 0.93)	0.00	-0.50 (-2.07, 1.06)	-0.22 (-1.07, 0.63)	-0.08 (-1.34, 1.19)	0.33
SF	0.00	-0.38 (-1.02, 0.26)	-0.14 (-0.63, 0.35)	-0.77 (-1.33, -0.21)**	0.00	0.40 (-0.76, 1.55)	-0.23 (-0.86, 0.40)	-0.48 (-1.41, 0.46)	0.99

NONS = neither obesity nor sarcopenia, IO = isolated obesity, IS = isolated sarcopenia, SO = sarcopenic obesity, PCS = physical component summary, MCS = mental component summary, GH = general health, PF = physical functioning, RP = role limitation due to physical problems, BP = bodily pain, RE = role limitation due to emotional problem, MH = mental health, VT = vitality, SF = social functioning

Adjusted β (95% CI): adjusted for sex, age, family income, education, occupation, smoking status, alcohol use, DASH diet adherence, snoring, physical activity, number of co-morbidities, number of falls in past 6 months, and GDS-15 score

*: *P* < 0.05, **: *P* < 0.01, ***: *P* < 0.001

Sensitivity analyses using four alternative SO criteria showed similar results for the association of SO with QoL (Supplementary Fig. 1–4), with no significant sex interaction observed (Supplementary Tables 2–5).

## Discussion

In this large population-based study, we found that SO was significantly associated with lower QoL in middle-aged to older adults, particularly in domains related to physical functioning, general health and bodily pain. However, no significant association was found between SO and mental health-related QoL, indicating that the detrimental effects of SO may be more confined to physical rather than psychological domains. These findings contribute to the growing body of evidence highlighting the distinct and severe burden of SO on physical health, particularly among middle-aged and older adults.

Previous studies examining the association between SO and QoL showed generally consistent results when using participants with NONS or those without sarcopenia as reference groups [9–11]. For example, a cross-sectional study on 95 Spanish women aged over 70 years showed significantly lower QoL scores (measured by SarQol, a sarcopenia-specific QoL scale) in women with SO than those without sarcopenia (mean [SD] SarQol overall score: 45.09 [7.9] vs. 66.51 [16.17], *P* = 0.001) [9]. In this study, SO was defined using a combination of muscle strength criteria (grip strength < 16 kg or chair stand test ≥ 17 s), and body composition measures (total skeletal muscle mass adjusted by weight ≤ 27.6% or fat mass > 40.9%). However, this study did not adjust for important potential confounders such as smoking status, alcohol use, and sleep duration, which may lead to biased estimates [9]. Similarly, another cross-sectional study involving 423 Turkish adults aged over 65 years found that SO was negatively associated VT score, as measured by the SF-36 (OR_SO vs. NONS_ = 0.96, 95% CI = 0.93 to 0.99) [10]. In this study, SO was defined using criteria that included BMI (BMI ≥ 30 kg/m^2^), skeletal muscle mass index (< 9.2 kg/m^2^ for men, < 7.4 kg/m^2^ for women), and indicators of physical function such as walking speed (< 0.8 m/s) and/or muscle strength (< 32 kg for men, < 22 kg for women) [10]. Furthermore, a large cross-sectional study on 11,521 Koreans aged over 20 years showed that SO was associated with lower scores of QoL on the EQ-5D scale, particularly in domains related to self-management, daily activity, and exercise ability, when compared to NONS [11]. SO in this study was defined based on the ratio of appendicular skeletal muscle to weight (%) (< 30.3 for men, < 23.8 for women) and WC (≥ 90 cm for men, ≥ 85 cm for women) [11]. These studies above used various definitions of SO, yet consistently demonstrated its detrimental impact on QoL. Our results align with these studies and add to the literature by providing robust estimates using a larger, well-characterized sample of middle-aged and older Chinese adults.

We also found that participants with SO had significantly lower physical health-related QoL than IO or IS. Evidence regarding the association of SO with QoL using obesity or sarcopenia alone as the reference is sparse. Only one cross-sectional study on 130 Lebanese aged over 18 years reported that SO was significantly associated with poorer QoL compared obesity alone [12]. Additionally, two studies that used sarcopenia alone as the reference reported mixed findings [10, 13]. A cross-sectional study in Turkey found no difference in QoL between participants with SO and those with sarcopenia alone [10]. However, a longitudinal study involving 261 hemodialysis patients reported that SO was associated with better QoL than sarcopenia alone [13]. However, these studies were limited by small sample sizes and involved samples that differ significantly from the general community [10, 12, 13].

Despite the current inconsistency in the definition of SO, we found similar results for the association of SO with QoL in sensitivity analyses using four alternative SO criteria (Supplementary Table 1). However, all the criteria of obesity were based on BMI or WC. Given the potentially nuanced and more complex changes in body composition in the aging population, future studies using other measures such as dual-energy X-ray absorptiometry (DXA), or bioelectrical impedance analysis (BIA) are warranted. Additionally, given the lack of clarity in categorizing severity, we did not assess the heterogeneity within the SO phenotype.

The mechanisms underlying the association of SO with QoL remain unclear, although several hypotheses have been proposed. First, SO is associated with increased fat deposition in muscle cells, which reduces the number of mitochondria and increases the production of reactive oxygen species. This process impairs muscle function and hinders the growth of new muscle tissue [1]. Furthermore, the inflammatory response and insulin resistance associated with SO may exacerbate loss of muscle mass and increase fat accumulation, further impairing muscle function and reducing health-related QoL [2, 28]. Among older adults, these factors also lead to difficulties in exercise, further deteriorating muscle function and mass, and reducing physical health-related QoL [29]. Moreover, the loss of muscle mass and strength in SO may increase the risk of osteoarthritis, which can further limit physical activity and negatively impact both physical and mental health [30, 31]. Inflammation associated with obesity or sarcopenia may also disrupt the hypothalamic-pituitary-adrenal (HPA) axis, potentially leading to depression [32]. However, this inflammation pathway appears to be more pronounced in younger individuals, who are more susceptible to metabolic disturbance such as inflammation and insulin resistance [32]. The lack of significantly association between SO and mental aspects of QoL in our study of middle-aged and older Chinese aligns with this observation.

The strengths of our study included the population-based design, the use of different definitions of SO, and the comprehensive adjustment for potential confounders. However, there were several limitations in our study. First, the causal relationship between SO and QoL could not be established in this cross-sectional study. Second, the overrepresentation of women in our sample may limit the generalizability of our findings to the broader population. However, given that no significant sex interaction was observed on QoL and adjustments for sex were made, this imbalanced is unlikely to have substantially affected our conclusions. Third, although we adjusted for multiple potential confounders in linear regression models, residual confounding could not be completely ruled out. In particular, while we accounted for the number of chronic conditions, the absence of a validated comorbidity index, such as the Charlson Comorbidity Index, may limit our ability to fully capture the impact of disease burden on QoL. Additionally, complex interactions among confounders that may nonlinearly affect QoL were not explicitly modeled. Further randomized controlled trials could provide more definitive evidence regarding causality and the effectiveness of specific interventions aimed at reducing SO and improving QoL.

## Conclusions

In conclusion, we found that SO was associated with lower physical health-related QoL compared to obesity or sarcopenia alone, but non-significantly associated with mental health-related QoL.

## Electronic supplementary material

Below is the link to the electronic supplementary material.

Supplementary Material 1

## Data Availability

Ethical approval in place allows us to share data on request. Please direct such requests to the Guangzhou Biobank Cohort Study Data Access Committee (gbcsdata@hku.hk).
