# Supplementary material for: Association between sarcopenic obesity, obesity, sarcopenia and quality of life in middle-aged and older Chinese: the Guangzhou Biobank Cohort Study
Source: Qual Life Res. 2025 Apr 2;34(7):1995–2004. doi: 10.1007/s11136-025-03960-9 (PMC12182485; doi:10.1007/s11136-025-03960-9)
Supplement: Supplementary file 1 — Supplementary Material 1 [file 11136_2025_3960_MOESM1_ESM.docx]

Supplementary Table 1 Criteria and prevalence for probable sarcopenia, obesity and sarcopenic obesity among 6,332 participants of the Guangzhou Biobank Cohort Study

| Criteria | N (%) |
| --- | --- |
| Probable sarcopenia (AWGS) | 3,842 (60.7) |
| 1): grip strength < 28 kg (men), < 18 kg (women) | 578 (9.1) |
| or 2): timed up-and-go test (TUGT) > 5s | 2,222 (35.1) |
| or 3): 1) + 2) | 1,042 (16.5) |
| Obesity |  |
| 1): BMI ≥ 28 kg/m^2^ | 680 (10.7) |
| 2): BMI ≥ 30 kg/m^2^ | 248 (3.9) |
| 3): WC ≥ 90 cm (men), 80 cm (women) | 1,985 (31.4) |
| Sarcopenic obesity |  |
| 1): Probable sarcopenia + obesity 1) or obesity 3) | 1,389 (21.9) |
| 2): Probable sarcopenia + obesity 2) or obesity 3) | 1,347 (21.3) |
| 3): Probable sarcopenia + obesity 1) | 487 (7.7) |
| 4): Probable sarcopenia + obesity 2) | 188 (3.0) |
| 5): Probable sarcopenia (base on grip strength only) + obesity 1) or obesity 3) | 525 (8.3) |

AWGS = Asian Working Group for Sarcopenia, TUGT = timed up-and-go test, WC = waist circumference.

**
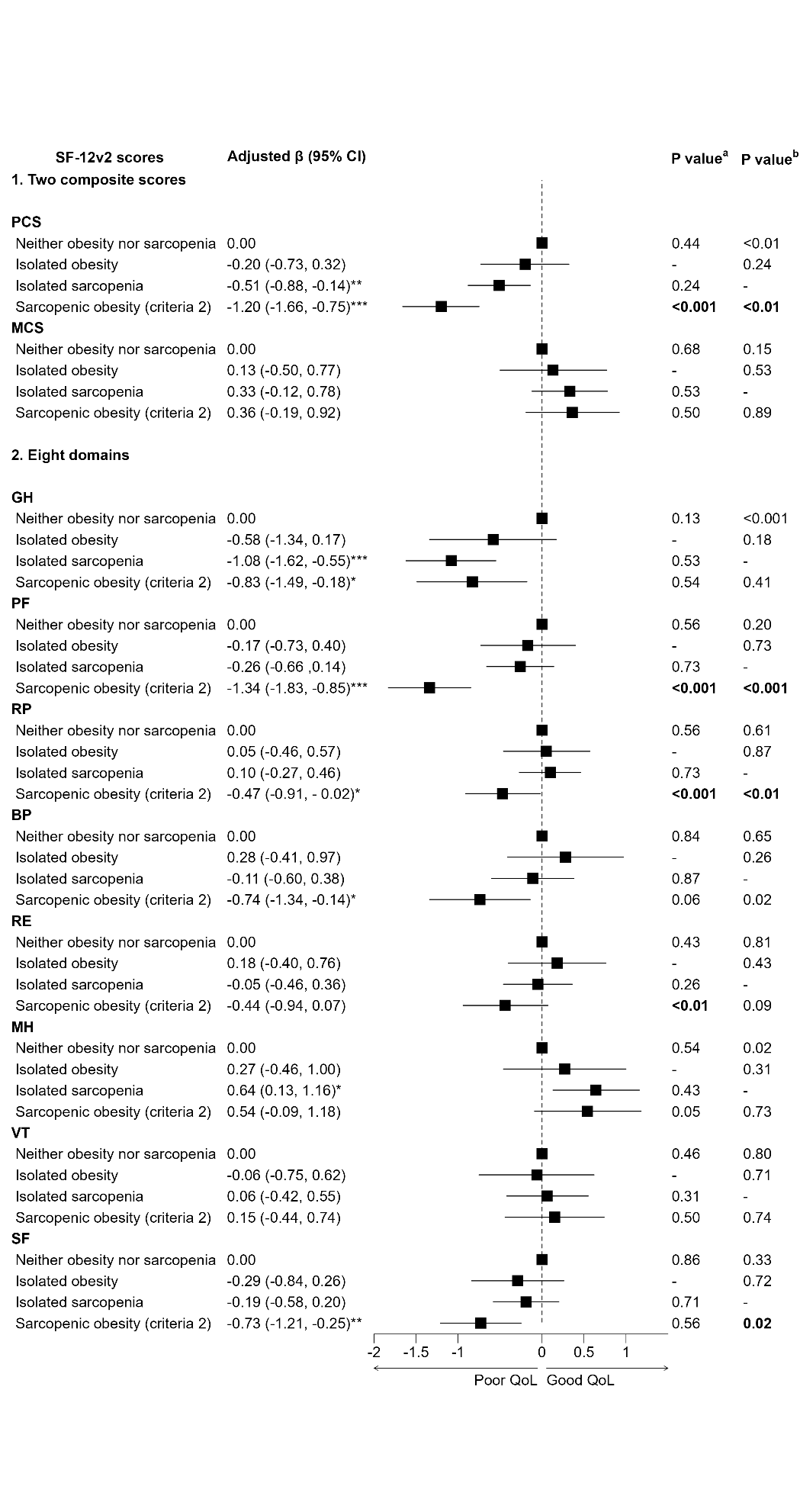
Supplementary Fig. 1 Association of sarcopenic obesity (criteria 2) with quality of life composite and domain scores on 6,332 participants of the Guangzhou Biobank Cohort Study**

Note: PCS = physical component summary, MCS = mental component summary, GH = general health, PF = physical functioning, RP = role limitation due to physical problems, BP = bodily pain, RE = role limitation due to emotional problem, MH = mental health, VT = vitality, SF = social functioning.

Adjusted β (95% CI): adjusted for sex, age, family income, education, occupation, smoking status, alcohol use, DASH diet pattern, snoring, physical activity, number of co-morbidities number of falls in past 6 months, and GDS-15 score, with neither obesity nor sarcopenia as the reference.

*: P <0.05, **: P < 0.01, ***: P < 0.001

a: with the isolated obesity as the reference

b: with the isolated sarcopenia as the reference

**
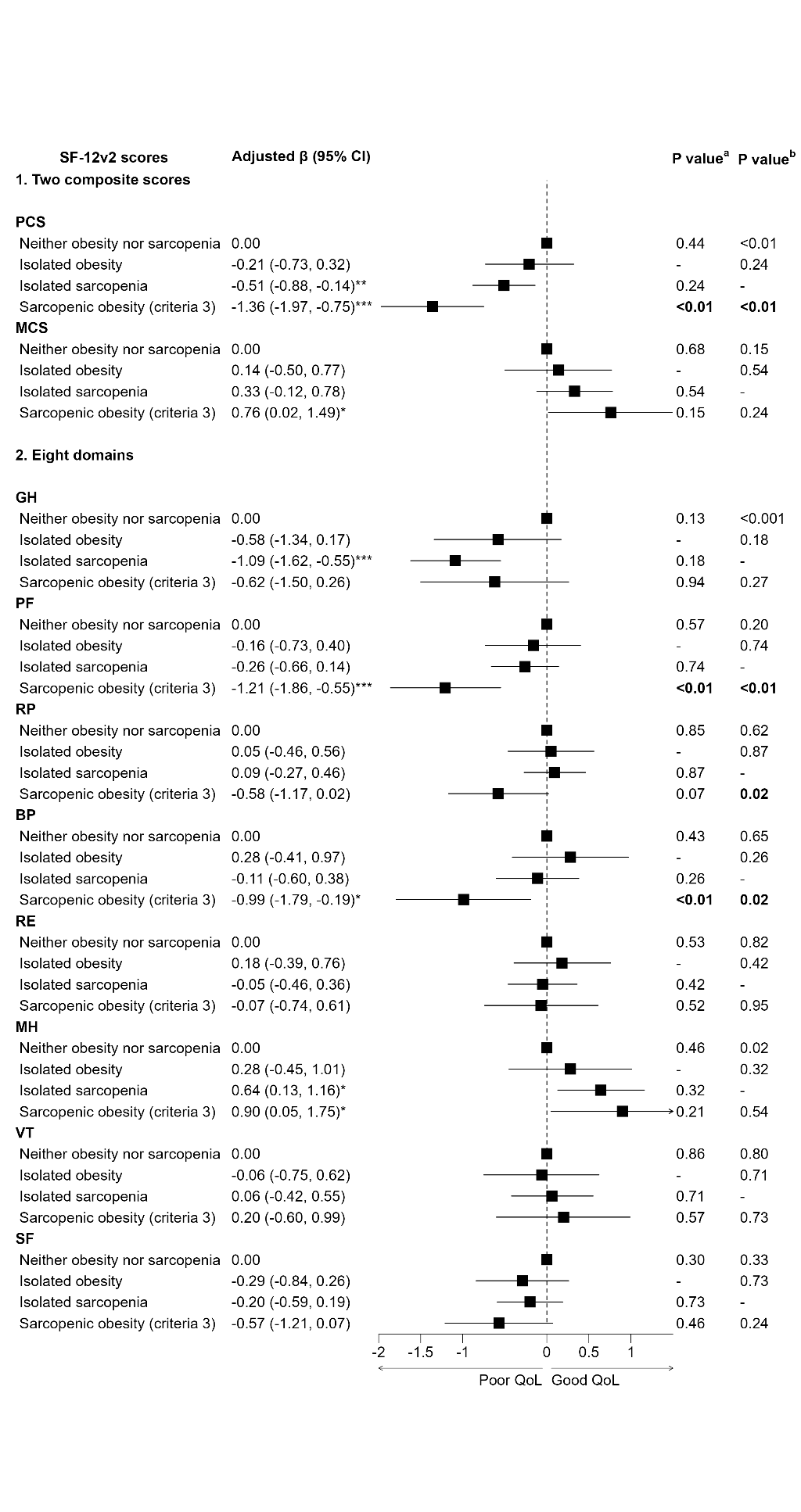
Supplementary Fig. 2 Association of sarcopenic obesity (criteria 3) with quality of life composite and domain scores on 6,332 participants of the Guangzhou Biobank Cohort Study**

Note: PCS = physical component summary, MCS = mental component summary, GH = general health, PF = physical functioning, RP = role limitation due to physical problems, BP = bodily pain, RE = role limitation due to emotional problem, MH = mental health, VT = vitality, SF = social functioning.

Adjusted β (95% CI): adjusted for sex, age, family income, education, occupation, smoking status, alcohol use, DASH diet pattern, snoring, physical activity, number of co-morbidities number of falls in past 6 months, and GDS-15 score, with neither obesity nor sarcopenia as the reference.

*: *P* <0.05, **: *P* < 0.01, ***: *P* < 0.001

a: with the isolated obesity as the reference

b: with the isolated sarcopenia as the reference

**
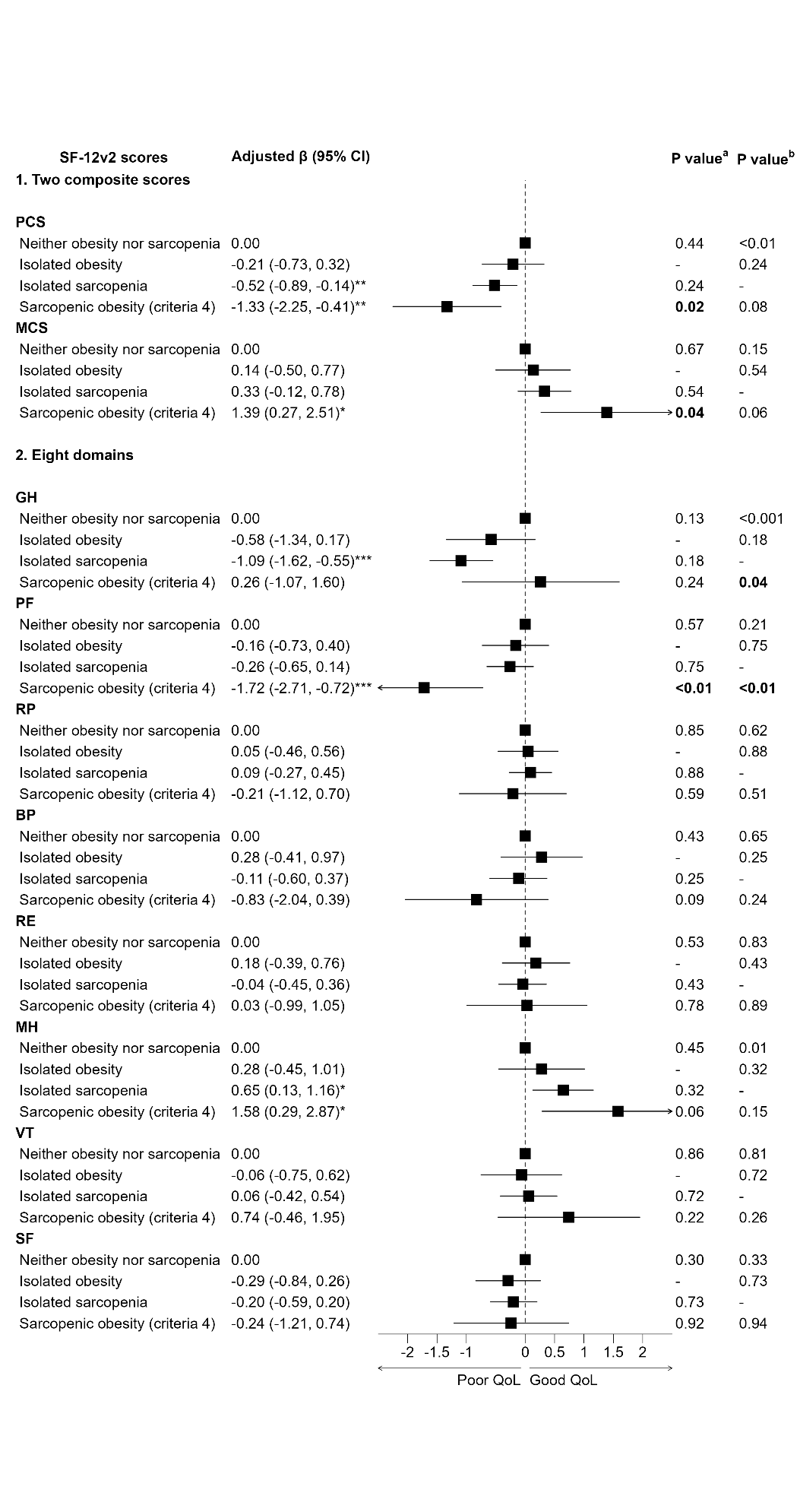
****Supplementary Fig. 3 Association of sarcopenic obesity (criteria 4) with quality of life composite and domain scores on 6,332 participants of the Guangzhou Biobank Cohort Study**

Note: PCS = physical component summary, MCS = mental component summary, GH = general health, PF = physical functioning, RP = role limitation due to physical problems, BP = bodily pain, RE = role limitation due to emotional problem, MH = mental health, VT = vitality, SF = social functioning.

Adjusted β (95% CI): adjusted for sex, age, family income, education, occupation, smoking status, alcohol use, DASH diet pattern, snoring, physical activity, number of co-morbidities number of falls in past 6 months, and GDS-15 score, with neither obesity nor sarcopenia as the reference.

*: *P* < 0.05, **: *P* < 0.01, ***: *P* < 0.001

a: with the isolated obesity as the reference

b: with the isolated sarcopenia as the reference


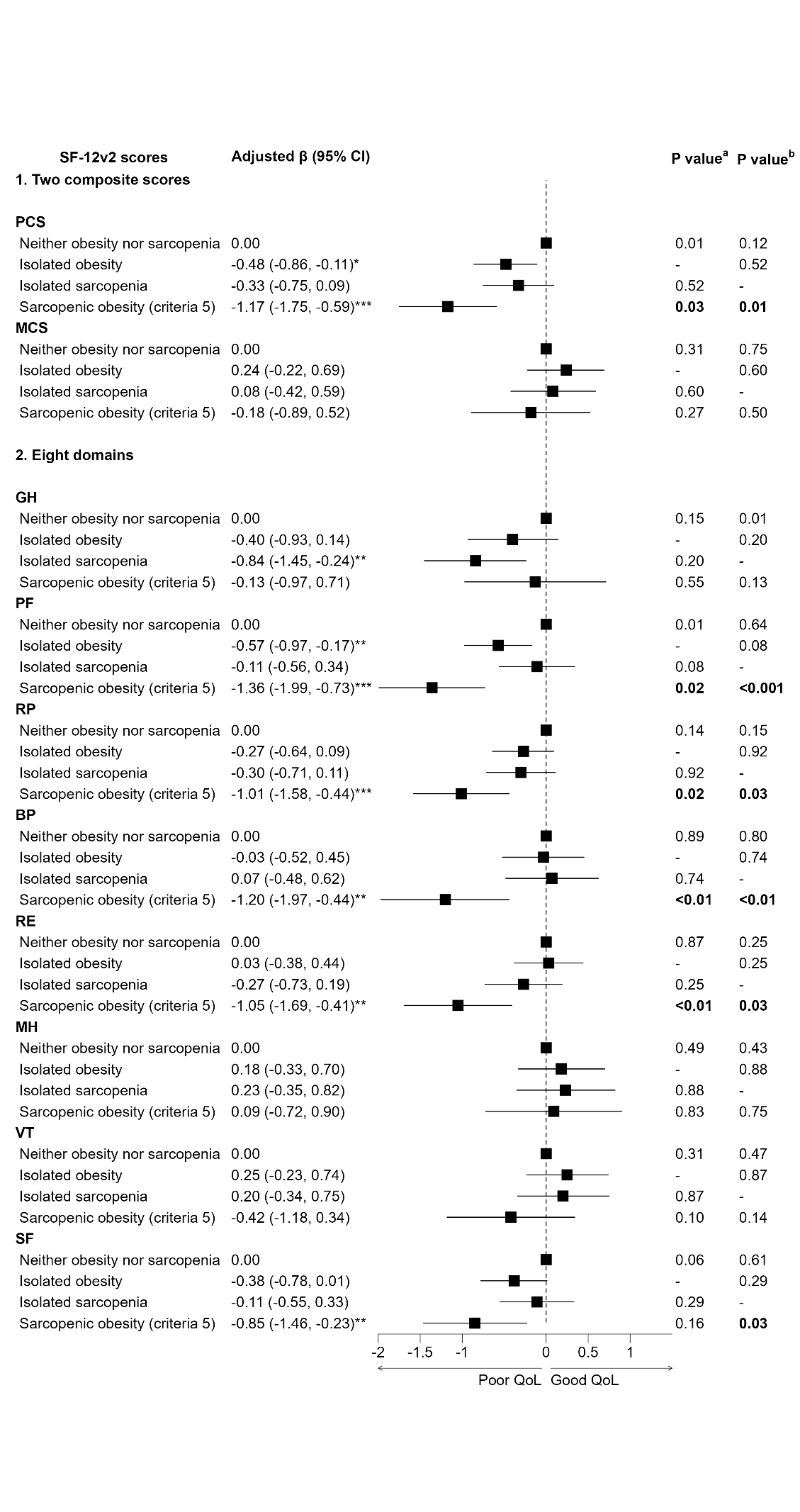
**Supplementary Fig. 4 Association of sarcopenic obesity (criteria 5) with quality of life composite and domain scores on 6,332 participants of the Guangzhou Biobank Cohort Study**

Note: PCS = physical component summary, MCS = mental component summary, GH = general health, PF = physical functioning, RP = role limitation due to physical problems, BP = bodily pain, RE = role limitation due to emotional problem, MH = mental health, VT = vitality, SF = social functioning.

Adjusted β (95% CI): adjusted for sex, age, family income, education, occupation, smoking status, alcohol use, DASH diet pattern, snoring, physical activity, number of co-morbidities number of falls in past 6 months, and GDS-15 score, with neither obesity nor sarcopenia as the reference.

*: *P* < 0.05, **: *P* < 0.01, ***: *P* < 0.001

a: with the isolated obesity as the reference

b: with the isolated sarcopenia as the reference

Supplementary Table 2 Associations of sarcopenic obesity (Criteria 2) with quality of life composite and domain scores in 4,771 women and 1,561 men of the Guangzhou Biobank Cohort Study

|  | Women, adjusted β (95% CI) | | | | Men, adjusted β (95% CI) | | | | *P* for interaction |
| --- | --- | --- | --- | --- | --- | --- | --- | --- | --- |
|  | NONS | IO | IS | SO (criteria 2) | NONS | IO | IS | SO (criteria 2) |  |
| Two composite scores | | | | | | | | | |
| PCS | 0.00 | -0.24  (-0.84, 0.37) | -0.54  (-1.00, -0.08)* | -1.16  (-1.69, -0.62)*** | 0.00 | -0.17  (-1.27, 0.93) | -0.50  (-1.10, 0.10) | -1.42  (-2.33, -0.51)** | 0.67 |
| MCS | 0.00 | 0.31  (-0.42, 1.03) | 0.56  (0.01, 1.12)* | 0.47  (-0.17, 1.11) | 0.00 | -0.21  (-1.62, 1.19) | -0.10  (-0.86, 0.67) | 0.50  (-0.67, 1.67) | 0.41 |
| Eight domains | | | | | | | | | |
| GH | 0.00 | -0.43  (-1.26, 0.41) | -0.88  (-1.52, -0.24)** | -0.69  (-1.43, 0.05) | 0.00 | -1.09  (-2.92, 0.74) | -1.51  (-2.51, -0.52)** | -1.36  (-2.88, 0.15) | 0.23 |
| PF | 0.00 | -0.27  (-0.93, 0.39) | -0.27  (-0.78, 0.24) | -1.43  (-2.02, -0.85)*** | 0.00 | 0.18  (-0.93, 1.30) | -0.27  (-0.87, 0.34) | -0.95  (-1.88, -0.02)* | 0.23 |
| RP | 0.00 | 0.04  (-0.55, 0.64) | 0.15  (-0.31, 0.60) | -0.30  (-0.83, 0.22) | 0.00 | 0.17  (-0.91, 1.25) | -0.05  (-0.64, 0.54) | -0.99  (-1.88, -0.09)* | 0.67 |
| BP | 0.00 | 0.40  (-0.41, 1.21) | -0.14  (-0.76, 0.48) | -0.64  (-1.36, 0.07) | 0.00 | -0.33  (-1.70, 1.05) | -0.06  (-0.80, 0.69) | -0.77  (-1.91, 0.37) | 0.48 |
| RE | 0.00 | 0.24  (-0.43, 0.91) | 0.04  (-0.47, 0.56) | -0.26  (-0.86, 0.33) | 0.00 | 0.08  (-1.12, 1.27) | -0.20  (-0.85, 0.45) | -0.79  (-1.79, 0.20) | 0.53 |
| MH | 0.00 | 0.53  (-0.30, 1.36) | 0.96  (0.33, 1.59)** | 0.60  (-0.13, 1.32) | 0.00 | -0.45  (-2.10, 1.20) | 0.02  (-0.88, 0.91) | 1.09  (-0.28, 2.46) | 0.81 |
| VT | 0.00 | 0.10  (-0.67, 0.87) | 0.20  (-0.39, 0.79) | 0.25  (-0.43, 0.93) | 0.00 | -0.50  (-2.07, 1.06) | -0.22  (-1.08, 0.63) | -0.08  (-1.38, 1.22) | 0.36 |
| SF | 0.00 | -0.38  (-1.02, 0.26) | -0.14  (-0.63, 0.35) | -0.78  (-1.34, -0.21)** | 0.00 | 0.39  (-0.76, 1.55) | -0.23  (-0.85, 0.40) | -0.37  (-1.33, 0.58) | 0.87 |

NONS = neither obesity nor sarcopenia, IO = isolated obesity, IS = isolated sarcopenia, SO = sarcopenic obesity, PCS = physical component summary, MCS = mental component summary, GH = general health, PF = physical functioning, RP = role limitation due to physical problems, BP = bodily pain, RE = role limitation due to emotional problem, MH = mental health, VT = vitality, SF = social functioning.

Adjusted β (95% CI): adjusted for sex, age, family income, education, occupation, smoking status, alcohol use, DASH diet pattern, snoring, physical activity, number of co-morbidities, number of falls in past 6 months, and GDS-15 score.

*: *P* < 0.05, **: *P* < 0.01, ***: *P* < 0.001

Supplementary Table 3 Associations of sarcopenic obesity (Criteria 3) with quality of life composite and domain scores in 4,771 women and 1,561 men of the Guangzhou Biobank Cohort Study

|  | Women, adjusted β (95% CI) | | | | Men, adjusted β (95% CI) | | | | *P* for interaction |
| --- | --- | --- | --- | --- | --- | --- | --- | --- | --- |
|  | NONS | IO | IS | SO (criteria 3) | NONS | IO | IS | SO (criteria 3) |  |
| Two composite scores | | | | | | | | | |
| PCS | 0.00 | -0.24  (-0.85, 0.37) | -0.54  (-1.00, -0.07)* | -1.63  (-2.34, -0.92)*** | 0.00 | -0.17  (-1.26, 0.93) | -0.50  (-1.10, 0.09) | -0.13  (-1.36, 1.11) | 0.33 |
| MCS | 0.00 | 0.31  (-0.41, 1.03) | 0.57  (0.01, 1.12)* | 0.87  (0.02, 1.71)* | 0.00 | -0.21  (-1.61, 1.19) | -0.10  (-0.86, 0.67) | 0.69  (-0.88, 2.27) | 0.32 |
| Eight domains | | | | | | | | | |
| GH | 0.00 | -0.43  (-1.27, 0.40) | -0.89  (-1.53, -0.25) ** | -0.73  (-1.71, 0.25) | 0.00 | -1.09  (-2.92, 0.74) | -1.51  (-2.51, -0.52)** | 0.32  (-1.73, 2.37) | 0.36 |
| PF | 0.00 | -0.27  (-0.93, 0.40) | -0.26  (-0.77, 0.25) | -1.54  (-2.32, -0.76)*** | 0.00 | 0.18  (-0.93, 1.30) | -0.27  (-0.87, 0.34) | 0.15  (-1.10, 1.41) | 0.14 |
| RP | 0.00 | 0.04  (-0.55, 0.64) | 0.15  (-0.31, 0.60) | -0.56  (-1.26, 0.14) | 0.00 | 0.18  (-0.90, 1.26) | -0.06  (-0.65, 0.53) | -0.46  (-1.67, 0.75) | 0.85 |
| BP | 0.00 | 0.40  (-0.42, 1.21) | -0.13  (-0.76, 0.49) | -1.26  (-2.21, -0.31)** | 0.00 | -0.32  (-1.69, 1.05) | -0.07  (-0.81, 0.68) | 0.46  (-1.08, 2.00) | 0.25 |
| RE | 0.00 | 0.24  (-0.43, 0.92) | 0.05  (-0.47, 0.56) | 0.04  (-0.75, 0.83) | 0.00 | 0.08  (-1.12, 1.28) | -0.20  (-0.85, 0.45) | -0.07  (-1.42, 1.27) | 0.62 |
| MH | 0.00 | 0.53  (-0.29, 1.36) | 0.96  (0.33, 1.59)** | 0.92  (-0.05, 1.88) | 0.00 | -0.45  (-2.10, 1.20) | 0.02  (-0.88, 0.91) | 1.29  (-0.57, 3.14) | 0.64 |
| VT | 0.00 | 0.10  (-0.67, 0.87) | 0.20  (-0.39, 0.79) | 0.15  (-0.75, 1.05) | 0.00 | -0.50  (-2.06, 1.07) | -0.23  (-1.08, 0.62) | 0.50  (-1.25, 2.26) | 0.44 |
| SF | 0.00 | -0.38  (-1.02, 0.26) | -0.14  (-0.63, 0.35) | -0.69  (-1.44, 0.06) | 0.00 | 0.40  (-0.75, 1.55) | -0.23  (-0.86, 0.39) | 0.03  (-1.26, 1.33) | 0.85 |

NONS = neither obesity nor sarcopenia, IO = isolated obesity, IS = isolated sarcopenia, SO = sarcopenic obesity, PCS = physical component summary, MCS = mental component summary, GH = general health, PF = physical functioning, RP = role limitation due to physical problems, BP = bodily pain, RE = role limitation due to emotional problem, MH = mental health, VT = vitality, SF = social functioning.

Adjusted β (95% CI): adjusted for sex, age, family income, education, occupation, smoking status, alcohol use, DASH diet pattern, snoring, physical activity, number of co-morbidities, number of falls in past 6 months, and GDS-15 score.

*: *P* < 0.05, **: *P* < 0.01, ***: *P* < 0.001

Supplementary Table 4 Associations of sarcopenic obesity (Criteria 4) with quality of life composite and domain scores in 4,771 women and 1,561 men of the Guangzhou Biobank Cohort Study

|  | Women, adjusted β (95% CI) | | | | Men, adjusted β (95% CI) | | | | *P* for interaction |
| --- | --- | --- | --- | --- | --- | --- | --- | --- | --- |
|  | NONS | IO | IS | SO (criteria 4) | NONS | IO | IS | SO (criteria 4) |  |
| Two composite scores | | | | | | | | | |
| PCS | 0.00 | -0.24  (-0.85, 0.37) | -0.54  (-1.01, -0.08)* | -1.78  (-2.83, -0.73)*** | 0.00 | -0.18  (-1.27, 0.92) | -0.49  (-1.08, 0.11) | 1.01  (-1.10, 3.13) | 0.42 |
| MCS | 0.00 | 0.31  (-0.41, 1.04) | 0.57  (0.01, 1.12)* | 1.41  (0.17, 2.66)* | 0.00 | -0.21  (-1.62, 1.19) | -0.09  (-0.86, 0.67) | 2.11  (-0.60, 4.81) | 0.34 |
| Eight domains | | | | | | | | | |
| GH | 0.00 | -0.43  (-1.27, 0.40) | -0.90  (-1.54, -0.26)** | 0.04  (-1.40, 1.48) | 0.00 | -1.10  (-2.93, 0.72) | -1.50  (-2.49, -0.50)** | 1.88  (-1.64, 5.39) | 0.28 |
| PF | 0.00 | -0.27  (-0.93, 0.39) | -0.26  (-0.77, 0.25) | -2.27  (-3.42, -1.13)*** | 0.00 | 0.18  (-0.94, 1.29) | -0.26  (-0.86, 0.35) | 1.15  (-0.99, 3.30) | 0.16 |
| RP | 0.00 | 0.04  (-0.55, 0.64) | 0.14  (-0.31, 0.60) | -0.31  (-1.33, 0.72) | 0.00 | 0.17  (-0.91, 1.25) | -0.05  (-0.64, 0.54) | 0.60  (-1.48, 2.67) | 0.83 |
| BP | 0.00 | 0.40  (-0.42, 1.21) | -0.14  (-0.76, 0.48) | -1.29  (-2.69, 0.11) | 0.00 | -0.33  (-1.70, 1.04) | -0.05  (-0.80, 0.70) | 1.93  (-0.71, 4.57) | 0.32 |
| RE | 0.00 | 0.24  (-0.43, 0.92) | 0.05  (-0.47, 0.57) | -0.03  (-1.19, 1.13) | 0.00 | 0.07  (-1.12, 1.27) | -0.19  (-0.84, 0.46) | 1.01  (-1.30, 3.31) | 0.70 |
| MH | 0.00 | 0.53  (-0.29, 1.36) | 0.96  (0.33, 1.59)** | 1.52  (0.09, 2.94)* | 0.00 | -0.45  (-2.10, 1.20) | 0.02  (-0.88, 0.92) | 2.78  (-0.39, 5.96) | 0.63 |
| VT | 0.00 | 0.10  (-0.67, 0.87) | 0.20  (-0.39, 0.79) | 0.57  (-0.76, 1.90) | 0.00 | -0.50  (-2.07, 1.06) | -0.22  (-1.07, 0.63) | 1.85  (-1.16, 4.85) | 0.40 |
| SF | 0.00 | -0.38  (-1.02, 0.26) | -0.14  (-0.63, 0.35) | -0.48  (-1.59, 0.63) | 0.00 | 0.40  (-0.75, 1.55) | -0.22  (-0.85, 0.40) | 1.63  (-0.58, 3.85) | 0.86 |

NONS = neither obesity nor sarcopenia, IO = isolated obesity, IS = isolated sarcopenia, SO = sarcopenic obesity, PCS = physical component summary, MCS = mental component summary, GH = general health, PF = physical functioning, RP = role limitation due to physical problems, BP = bodily pain, RE = role limitation due to emotional problem, MH = mental health, VT = vitality, SF = social functioning.

Adjusted β (95% CI): adjusted for sex, age, family income, education, occupation, smoking status, alcohol use, DASH diet pattern, snoring, physical activity, number of co-morbidities, number of falls in past 6 months, and GDS-15 score.

*: *P* < 0.05, **: *P* < 0.01, ***: *P* < 0.001

Supplementary Table 5 Associations of sarcopenic obesity (Criteria 5) with quality of life composite and domain scores in 4,771 women and 1,561 men of the Guangzhou Biobank Cohort Study

|  | Women, adjusted β (95% CI) | | | | Men, adjusted β (95% CI) | | | | *P* for interaction |
| --- | --- | --- | --- | --- | --- | --- | --- | --- | --- |
|  | NONS | IO | IS | SO (criteria 5) | NONS | IO | IS | SO (criteria 5) |  |
| Two composite scores | | | | | | | | | |
| PCS | 0.00 | -0.49 | -0.44 | -1.16 | 0.00 | -0.62 | -0.30 | -1.09 | 0.37 |
|  |  | (-0.92, -0.06) * | (-0.96, 0.08) | (-1.81, -0.50) *** |  | (-1.38, 0.14) | (-0.99, 0.39) | (-2.47, 0.30) |  |
| MCS | 0.00 | 0.31 | 0.16 | -0.39 | 0.00 | 0.08 | 0.13 | 1.27 | 0.76 |
|  |  | (-0.20, 0.83) | (-0.47, 0.78) | (-1.17, 0.39) |  | (-0.89, 1.05) | (-0.75, 1.01) | (-0.50, 3.04) |  |
| Eight domains | | | | | | | | | |
| GH | 0.00 | -0.35 | -0.80 | -0.24 | 0.00 | -0.72 | -0.97 | 0.42 | 0.82 |
|  |  | (-0.94, 0.25) | (-1.51, -0.08) * | (-1.14, 0.66) |  | (-1.98, 0.55) | (-2.11, 0.18) | (-1.89, 2.73) |  |
| PF | 0.00 | -0.67 | -0.04 | -1.37 | 0.00 | -0.16 | -0.38 | -1.19 | 0.77 |
|  |  | (-1.15, -0.20) ** | (-0.61, 0.53) | (-2.09, -0.65) *** |  | (-0.94, 0.61) | (-1.08, 0.32) | (-2.60, 0.22) |  |
| RP | 0.00 | -0.19 | -0.36 | -0.99 | 0.00 | -0.71 | -0.35 | -0.64 | 0.39 |
|  |  | (-0.62, 0.23) | (-0.87, 0.15) | (-1.63, -0.35) ** |  | (-1.46, 0.03) | (-1.02, 0.33) | (-2.00, 0.72) |  |
| BP | 0.00 | 0.03 | -0.28 | -1.29 | 0.00 | -0.40 | 0.73 | -0.34 | 0.05 |
|  |  | (-0.54, 0.61) | (-0.97, 0.42) | (-2.17, -0.42) ** |  | (-1.35, 0.54) | (-0.13, 1.59) | (-2.07, 1.39) |  |
| RE | 0.00 | 0.19 | -0.22 | -1.14 | 0.00 | -0.51 | -0.27 | 0.05 | 0.60 |
|  |  | (-0.29, 0.67) | (-0.80, 0.36) | (-1.87, -0.42) ** |  | (-1.33, 0.32) | (-1.02, 0.48) | (-1.46, 1.56) |  |
| MH | 0.00 | 0.17 | 0.27 | -0.11 | 0.00 | 0.40 | 0.28 | 1.47 | 0.61 |
|  |  | (-0.42, 0.76) | (-0.43, 0.98) | (-1.01, 0.78) |  | (-0.74, 1.54) | (-0.75, 1.31) | (-0.62, 3.55) |  |
| VT | 0.00 | 0.34 | 0.30 | -0.41 | 0.00 | -0.00 | 0.06 | -0.40 | 0.53 |
|  |  | (-0.21, 0.89) | (-0.37, 0.96) | (-1.24, 0.42) |  | (-1.08, 1.08) | (-0.91, 1.04) | (-2.37, 1.57) |  |
| SF | 0.00 | -0.41 | -0.16 | -1.15 | 0.00 | -0.27 | 0.07 | 0.94 | 0.15 |
|  |  | (-0.87, 0.05) | (-0.71, 0.39) | (-1.84, -0.45) ** |  | (-1.06, 0.53) | (-0.65, 0.79) | (-0.51, 2.40) |  |

NONS = neither obesity nor sarcopenia, IO = isolated obesity, IS = isolated sarcopenia, SO = sarcopenic obesity, PCS = physical component summary, MCS = mental component summary, GH = general health, PF = physical functioning, RP = role limitation due to physical problems, BP = bodily pain, RE = role limitation due to emotional problem, MH = mental health, VT = vitality, SF = social functioning.

Adjusted β (95% CI): adjusted for sex, age, family income, education, occupation, smoking status, alcohol use, DASH diet pattern, snoring, physical activity, number of co-morbidities, number of falls in past 6 months, and GDS-15 score.

*: *P* < 0.05, **: *P* < 0.01, ***: *P* < 0.001
